# A key to the *Sabdariffa scotellii* (Baker f.) Mwachala & R.L.Barrett group (Malvaceae, Hibisceae) in West Africa with descriptions of two new, threatened species from bowal habitats in the Republic of Guinea

**DOI:** 10.3897/phytokeys.271.181271

**Published:** 2026-02-10

**Authors:** Aminata Thiam, Sekou Magassouba, Charlotte Couch, Martin Cheek

**Affiliations:** 1 Herbier National de Guineé, Université de Gamal Abdel Nasser, Conakry, BP 680, Guinea West Africa Plant Red List Authority, IUCN Species Survival Commission Gland Switzerland; 2 Herbarium, Royal Botanic Gardens, Kew, Richmond, Surrey, TW9 3AE, UK Université de Gamal Abdel Nasser Conakry Guinea; 3 West Africa Plant Red List Authority, IUCN Species Survival Commission, Rue Mauverney 28, 1196 Gland, Switzerland Royal Botanic Gardens Kew United Kingdom

**Keywords:** Conservation, crops, extinction, Guinea Highlands, *
Hibiscus
*, Roselle

## Abstract

Two species new to science from the Republic of Guinea, West Africa are placed in the informal *Sabdariffa scotellii* group within *Sabdariffa* (formerly *Hibiscus* sect. *Furcaria*, Malvaceae). The group is characterized by the combination of entire (not bifurcate) involucellar bracts, non-aculeate stems, 3–5-lobed leaves abaxially with 1 or usually 3 nectar glands at the base of the midrib and flanking lateral nerves. An identification key to the four species of the group is presented. The new species are described in this paper as *Sabdariffa sangaredi* Thiam & Cheek, **sp. nov**. (from bauxitic low altitude bowal in the Sangaredi area) and *Sabdariffa kounounkan* Cheek, **sp. nov**. (from montane sandstone bowal on the Kounounkan table mountain). Their taxonomic affinities and ecology are discussed. Both species are assessed as Critically Endangered using the IUCN 2012 standard. We also discuss other recently described endemics of sandstone habitats in Guinea.

## Introduction

The flora of Guinea (Republic of Guinea) (245,857 km^2^) is diverse in a West African context. Lisowski (2009) details approximately 3,000 species and Gosline et al. (2023a, b) raises this number to nearly 4,000 indigenous and naturalised species. This includes numerous endemic species and five endemic genera; *Fleurydora* A. Chev. (Ochnaceae), *Cailliella* Jacq.-Fél., *Feliciadamia* Bullock, and *Benna* Burg & Ver.-Lib. (all Melastomataceae) and *Kindia* Cheek (Rubiaceae) (Gosline et al. (2023a, b).

Since 2005, botanical surveys for conservation management have been conducted by botanists of the Royal Botanic Gardens, Kew (K) and others (e.g. of the Meise Botanic Garden, Brussels and the Missouri Botanical Garden), with the National Herbarium of the Université Gamal Abdel Nasser, Conakry, Guinea (HNG). Additional species and genera new to science from Guinea have been published regularly (Goyder 2009; Champluvier and Darbyshire 2009; Fischer et al. 2011; Darbyshire et al. 2012; Cheek and van der Burgt 2010; van der Burgt et al. 2012; Prance and Jongkind 2015; Cheek and Haba 2016a, b; Cheek and Williams 2016; Cheek et al. 2016, 2019a, 2024a, b; Phillipson et al. 2019; van der Burgt et al. 2022; Jongkind 2023; Breteler and Baldé 2024).

The genus *Sabdariffa* (DC.) Kostel. (formerly *Hibiscus* Sect. *Furcaria* DC.) was recently formally segregated from *Hibiscus* L. (Barrett et al. 2025), more than 50 years since this action was mooted by Menzel and Wilson (1969). Unlike *Hibiscus* sensu stricto, most species of this very distinct genus bear a nectary gland on the abaxial surface of the leaf midrib and on the midrib of the sepals, and *Sabdariffa* is part of a clade of genera characterized by this trait, which is quite distinct from *Hibiscus* and its allies (Pfeil et al. 2002; Barrett et al. 2025). *Decaschistia* Wight & Arn., *Kydia* Roxb., *Talipariti* Fryxell, and *Urena* L. are placed in the same subclade as *Sabdariffa* (Hanes et al. 2024; Barrett et al. 2025). *Sabdariffa* is characterized by a fruiting calyx that is leathery to fleshy, with thickened, raised, rib-like veins both along the midrib of each sepal and from the receptacle to the notch of each sinus, continuing along the margin of the sepals. Most species also possess forked involucellar (epicalyx) bracts, from which the section *Furcaria* took its name, and aculeate stems, both features are present in about 2/3 of the African species. Most species of *Sabdariffa* in Africa also possess fruit valves which are abaxially setose.

*Sabdariffa* is pantropical, rarely extending into temperate areas, and currently has 117 species with two major centres of about equal species diversity; South America (43 spp.) and sub-Saharan Africa (42 spp.), followed by Australia (37 taxa). Only one species, *S. diversifolia* (Jacq.) McLay & R.L. Barrett, is considered indigenous in all three centres (Barrett et al. 2025, table 2). These totals include introduced taxa (respectively 6, 2, and 3). In Africa, the species mainly grow in open habitats in areas with < 2 m rainfall p.a. and are annuals, perennial herbs, shrubs, or climbers.

The genus *Sabdariffa* contains several commercially important and globally cultivated crop species, all indigenous to tropical Africa, such as kenaf used for fibre i.e. *S. cannabina* (L.) M.M. Hanes & R.L. Barrett (formerly *Hibiscus cannabinus* L.), bissap, hibiscus tea, or roselle with edible fleshy calyces used for making juices and tea (*S. gossypiifolia* (Mill.) M.M. Hanes & R.L. Barrett, formerly *H. sabdariffa* L.), and, used as a leaf vegetable, *S. acetosella* (Welw. & Hiern) M.M. Hanes & R.L. Barrett, (formerly *H. acetosella* Welw. ex Ficalho). Several other lesser known species in Africa are also grown for stem fibres and as a leaf vegetable, e.g. *S. radiata* (Cav.) R.L. Barrett & M.M. Hanes and *S. noldeae* (Baker f.) Mwachala & R.L. Barrett. However, there are also several rare, more range restricted species such as *S. sparseaculeta* (Baker f.) Mwachala & R.L. Barrett (Cheek 1992), which are not known to be used domestically.

Many African species are known only from the type collection (Wilson 1999), e.g. *Sabdariffa furcellatoides* (Hochr.) Mwachala & R.L. Barrett of Guinea, *S. moxicoensis* (Baker f.) Mwachala & R.L. Barrett and *S. flavorosea* (Baker f.) Mwachala & R.L. Barrett, both of Angola, *S. elongatifolia* (Hochr.) Mwachala & R.L. Barrett (Cameroon), *S. paolii* (Mattei) Mwachala & R.L. Barrett (Somalia), *S. goossensii* (Hauman) Mwachala & R.L. Barrett of Democratic Republic of the Congo, or are from single locations e.g. *S. cuanzensis* (Exell & Mendonça) Mwachala & R.L. Barrett (Angola). Several species are Red Listed as threatened, including the recently described *S. fabiana* (Cheek) Mwachala & R.L. Barrett (VU; Budden and Cheek 2023) and *S. ngokbanakii* (Burg) Mwachala & R.L. Barrett (EN; Ikabanga et al. (2020).

*Sabdariffa* (as *Hibiscus* section *Furcaria*), was expertly revised for Africa by Wilson (1999) who treated 33 species from this area. Barrett et al. (2025) revised and updated Wilson’s treatment, largely confirming his species delimitations, and adding subsequently described species, e.g. two new species from Gabon (van der Burg 2013), and one from W Africa (Cheek et al. 2020), raising the total number of taxa in Africa to 42 (Barrett et al. 2025).

Nine of the 13 genomes known in *Sabdariffa* are found in Africa, and Africa contains nine of the 10 known diploid species (Wilson 1999). In the diploid species, combinations of the major morphological characters (stems aculeate or not; peduncle articulated at base or near apex; involucellular bracts bifurcate or not, and calyx lobes with nectar gland or not) are correlated with their genomes (Wilson 1999).

Because our material lacks aculeate stems and bifurcate involucellar bracteoles, it keys out in Wilson (1999) to couplet 36 (and couplet 46 in Key 1 of Barrett et al. 2025), which both lead to the same four species, one of which is the smooth stemmed cultivar of *Sabdariffa gossypiifolia* (formerly *Hibiscus sabdariffa*) which is an unusual species in the genus owing to its entirely fleshy, edible calyx (see above). Since our material lacks this feature, and other diagnostic features of this distinctive species, we can rule this out as matching our specimens. A further species from couplet 36 that can be ruled out is *S. elongatifolia* (Hochr.) Mwachala & R.L. Barrett. This Cameroonian species, one of those known only from the type collection, differs from our species in that the stipules are persistent and divided into linear segments (not entire as is usual in the genus) and that the leaf-blade is not 3–5-lobed, but entire and linear, and lacks a nectar gland. The remaining two species, *S. scotellii* (Baker f.) Mwachala & R.L.Barrett and *S. sineaculeata* F.D. Wilson (the second relatively recently segregated from the first (Wilson 1999), that occur at couplet 38, are more similar to our two new taxa, forming a group here informally termed the *S. scotellii* group.

Here we describe two new species as *Sabdariffa sangaredi* Thiam & Cheek, sp. nov. and *S. kounounkan* Cheek, sp. nov. The specimens on which these are based match no other species or infra-specific taxa of *Sabdariffa*.

### The *Sabdariffa scotellii* group

We characterize the *Sabdariffa scotellii* species group by their entire involucellular bracteoles, non-aculeate stems, and also the palmately 3 or 5 (rarely 7-lobed) leaves. They also have nectar glands on the midribs of both the abaxial leaf blade and on the calyx lobes, and seed surfaces with either stellate hairs or transverse convex ellipsoid microstructures (lacking the pectinate scales that are usual in the genus). Because they have these characters, specimens of the new taxa were initially assigned to or flagged as having an affinity with *S. scotellii*.

Major sources of characters for classifying and delimiting taxa in *Sabdariffa* are the degree of lobing of the leaves, the indumentum complement of the stem, leaves, fruiting epicalyx and calyx, the characteristics of the nectar glands, and the seed ornamentation (Mwachala 1995). In contrast, the petals and sexual floral parts provide relatively few characters of taxonomic value at the species level.

## Materials and methods

Herbarium material was examined with a Leica Wild M8 dissecting binocular microscope fitted with an eyepiece graticule measuring in units of 0.025 mm at maximum magnification. The drawings were made with the same equipment with a Leica 308700 camera lucida attachment. Specimens, or their high-resolution images, of all African species of *Sabdariffa* (sensu Barrett et al. 2025) were inspected from the following herbaria: BM, HNG, K, P, and SL. All specimens cited have been seen. Specimens were collected using the patrol method and processed as in Davies et al. (2023). Names of species and authors follow the International Plant Names Index (IPNI 2025). Nomenclature follows Turland et al. (2025). Technical terms follow Beentje and Cheek (2003). The format of the descriptions follows those in other papers describing new species of *Sabdariffa* (e.g., S. *fabiana* as *H. fabiana*, Cheek et al. 2020). The conservation assessment follows the IUCN (2012) categories and criteria. Extent of occurrence was calculated using GeoCAT (Bachman and Moat 2012). Herbarium codes follow Index Herbariorum (Thiers 2025).

## Taxonomic treatment

### Key to the species of the *Sabdariffa scotellii* group, West Africa

**Table:** 

1	Abaxial leaf surface with nectar glands at base of 3 largest nerves; calyx intercostal areas minutely and softly hairy, lacking bristle hairs with fleshy red swollen bases; leaves 5(−7)-lobed	**2**
–	Abaxial leaf surface with one nectar gland at base of midrib only; calyx intercostal areas with bristle hairs with fleshy red swollen bases; leaves 3(−5)-lobed	**3**
2	Leaf-blades lobed by >9/10 their radius; marginal calyx nerves conspicuous; granite inselbergs	***Sabdariffa scotellii***
–	Leaf-blades lobed by <1/3 their radius; marginal calyx nerves inconspicuous; sandstone table mountains	***Sabdariffa kounounkan***
3	Stem without a longitudinal band of minute, soft, simple hairs; stem hairs stellate; calyx moderately densely stellate hairy; Ghana to Nigeria	***Sabdariffa sineaculeata***
–	Stem with a longitudinal band of minute, soft, simple hairs; stem hairs otherwise a mix of simple, 2-fid, 3-fid and stellate; calyx glabrous apart from sparse simple bristle hairs; Guinea	***Sabdariffa sangaredi***

#### Sabdariffa sangaredi

Taxon classificationPlantaeMalvalesMalvaceae

Thiam & Cheek sp. nov.

3534C70E-C015-550D-A444-60FD39042AB7

urn:lsid:ipni.org:names:77376560-1

Figs 1, 2

##### Type.

Republic of Guinea • Boké Prefecture, Sangaredi, Para Gogo, Bowal Dalagahe, 11°7'52.7"N, 013°51'10.6"W, 228 m elev., 19 Nov 2013 (fl, fr), *K. Guilavogui, L. Lopez Poveda & M.B. Bah 613* (holotype: K barcode K000749855; isotype: HNG).

##### Diagnosis.

Similar to *Sabdariffa sineaculeata* (F.D. Wilson) Mwachala and R.L. Barrett and *S. scotellii* (Baker f.) Mwachala and R.L. Barrett in the non-aculeate stems, entire involucellar bracts, non-fleshy calyx, palmately lobed leaves, nectar glands on leaves and calyx, differing in the involucellar bracts 3 mm wide (not 2 mm or less), the calyx with only simple broad-based bristle hairs (not stellate hairy), the leaves 3-lobed by c. 8/10^ths^ their radius (not shallowly 3-lobed by <1/3 or 5-lobed by >9/10^ths^).

**Figure 1.**
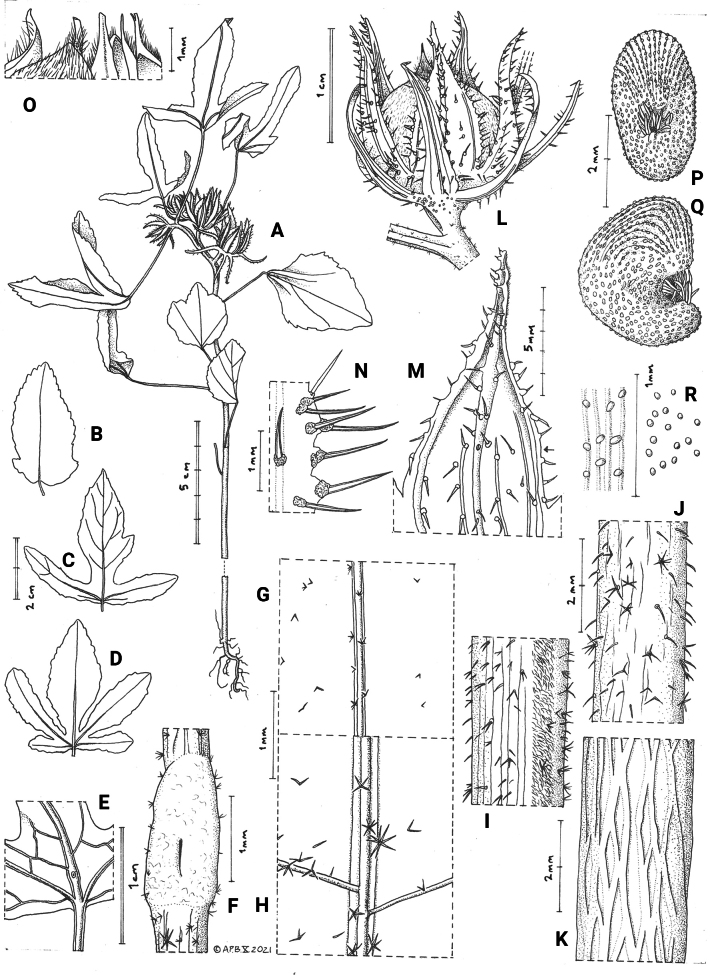
*Sabdariffa sangaredi* Thiam & Cheek, sp. nov. **A**. Habit, fruiting plant; **B–D**. Leaf shape variation; **E**. Base of leaf blade, abaxial surface showing nectar gland; **F**. Detail from E of nectar gland; **G**. Adaxial surface of leaf blade; **H**. Abaxial surface of leaf blade; **I**. Stem showing hair band; **J**. Upper stem; **K**. Base of stem; **L**. Post-anthetic flower/immature fruit; **M**. Calyx lobe showing minute nectar gland on midrib (see arrow); **N**. Detail of hairs with swollen bases on calyx margin; **O**. Apex of dehisced fruit valves; **P**. Seed, ventral view; **Q**. Seed, side view; **R**. Variation in seed surface sculpture. Drawn from *K. Guilavogui et al. 613* (K) by Andrew Brown.

##### Description.

***Annual herbs***, generally erect, (10−)20–35(−120) cm in height. ***Stem*** internodes 2–3.5 cm long, pale yellow, c. 2 mm in diam., lateral branches frequent in larger plants, ascending, 1–11.3 cm long, indumentum of distal 5–9 nodes sparsely hairy c. 10% cover, hairs soft, translucent or white, patent or retrorse, simple, bifid and trifid, (0.2−)0.3–0.4(−0.5) mm long, mixed with 4–7-armed stellate hairs 0.125–0.5 mm diam., the arms suberect or spreading and with minute red, sessile, globose glands 0.05 mm diam.; longitudinal hair band grey-brown, c. 0.4 mm wide, 80–100% cover of minute soft, simple and stellate subappressed, reflexed hairs 0.1–0.2 mm long; proximal (basal) nodes of stems glabrous with network of corky ridges. ***Stipules*** deciduous, subulate to linear-oblong, 0.75–1.75 × 0.25 mm, apex rounded, indumentum sparse, simple, translucent, stiff, appressed hairs 0.1–0.3 mm long. ***Petioles*** plano-convex, 1.5–4 cm long, 0.3–1 mm wide, indumentum as stem, adaxial surface with longitudinal hairband. ***Leaves*** on the main axis subtending flowers are deeply 3-lobed (very rarely 5-lobed), triangular in outline, 3–5.5 × 4.2–5(−8) cm, while the lower (proximal) leaves are entire, ovate, or elliptic-oblong, 2.5–5 × 2.2–3.5 cm, leaves on the lateral branches are slightly smaller; lobes oblong-elliptic, lateral lobes 2–4 × 0.5–0.8 cm, extending to 8/10^th^ of the radius of the blade, at c. 90 degrees from the median lobe, median lobe 10–20% longer and broader than the laterals, sometimes with a secondary lobe, lobes with 2–12 shallowly dentate to serrate teeth per side, teeth 0.1–0.3 mm long, apices red; secondary nerves conspicuous on both surfaces, reticulate, cells c. 2 mm diam.; nectar gland on the abaxial surface inserted on midrib c. 2 mm above the lateral nerve junction, gland 1.8 × 0.8 mm, broader than the midrib, the stoma longitudinal slit 0.5 mm long, glabrous apart from minute marginal, simple to stellate hairs c. 0.1 mm long, adaxial blade surface with very sparse (<5% cover) minute simple to stellate hairs c. 0.1 mm long, densest on midrib, abaxial surface as adaxial but cover and hair length slightly greater, stellate hairs 5–8-armed, 0.4 mm wide. ***Flowers*** yellow, with black adaxial markings (not dissected due to limited material). Peduncles-pedicels 0.4–1 × 0.25–3 mm, indumentum similar to that of the stem, not articulated. ***Involucellar bracts*** (epicalyx) 7–8, reflexing in mature fruit, dorsiventrally flattened, narrowly triangular, 1.25–1.6 × 0.3 cm wide at base, tapering gradually to the acute apex, united at base for c. 1 mm, midrib thickened, lateral vein pair parallel, abaxial surface glabrous apart from 6–12 patent bristle hairs c. 1 mm long. ***Calyx*** lobes 5, slightly longer than epicalyx, ovate triangular, 1.3–1.6 × 0.6 cm, acuminate, united at base for c. 2 mm, midrib and marginal nerves thick, prominent, with 0.9–1.1 mm long spreading or patent bristle hairs on thick red fleshy bases c. 0.25 × 0.25 mm, intercostal areas glabrous apart from a few bristle hairs on subsidiary nerves, abaxial surface glabrous apart from the densely puberulent acumen, hairs patent, c. 0.1 mm long. ***Capsules*** solitary, axillary, sometimes on a very short branch, borne on the distal nodes of the main stem and short lateral branches; fruit oblate, c. 1.5 × 1.2 cm, rostrum 0.7–2 mm long, abaxial surface densely covered in fine appressed simple hairs 0.2–0.4 mm long. ***Seeds*** subreniform, dull black, c. 3.25 × 1.8 × 2.5 mm, hilar/funicular cavity 0.75 × 0.75 mm, occluded by dense, patent, interlocking, broad white simple hairs 0.2–0.4(−0.5) mm long, outer surface glabrous, chalazal (cotyledonary) half with longitudinal ridges 0.1–0.2 mm apart, lined with brown transversely ellipsoid to globose smooth convex structures 0.06–0.12 mm long; micropyle (radicular) half lacking ridges, with globose black structures 0.05 mm diam.

**Figure 2.**
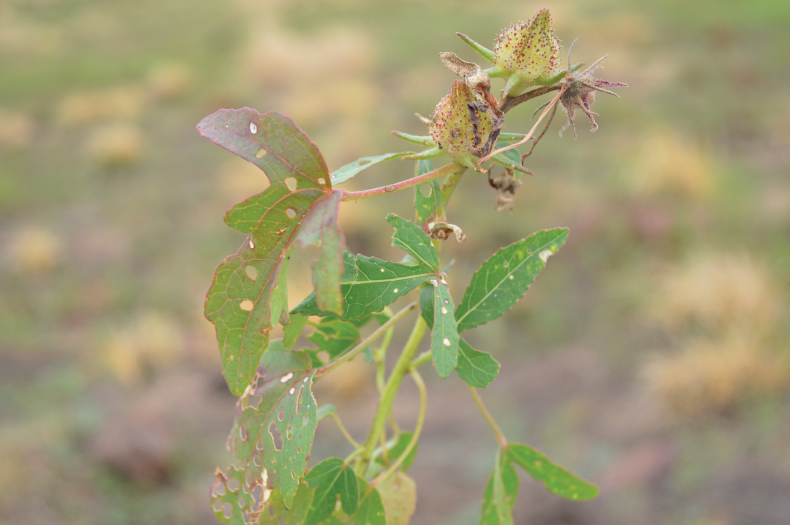
*Sabdariffa sangaredi* Thiam & Cheek, sp. nov. Photo from the field of *K. Guilavogui et al. 613* by Lucia Lopez Poveda.

##### Additional sight records.

Republic of Guinea • Boké Prefecture, Sangaredi, Kourawel, Feloparawol Aliou, N of Para Gogo, 11°9'9.3"N, 013°52'27"W, 24 Nov 2013, *F. Fofana & L. Lopez Poveda CBG Z7.1L03* (observation) • Fetore, W of Para Gogo, georeference not recorded, 21 Nov 2013, *K. Guilavogui & L. Lopez Poveda CBG Z6L04*. • c.7 km W of Kewel Bondi, 3.5 km northeast from Boulere, 11°9'32.6" N, 013°56'24.2"W, *C. Couch & F. Fofana CBG Z7C07*. • Haure diari de, 1.04 km of Kagnana, 11°0'15.7"N, 013°52'29.4"W, 23 Nov 2013, *K. Guilavogui & L. Lopez Poveda CBG Z5L02* • Konakrede forest, next to Murmutu bowal, 11°9'5.1"N, 013°56'37.2"W, 22 Nov 2013, *K. Guilavogui & L. Lopez Poveda CBG BOUL04* • Banire, 2 km of Kagnana, 11°0'49.9"N, 013°51'56.0"W, 23 Nov 2013, *K. Guilavogui & L. Lopez Poveda* CBG Z5L06 • Boulere, 0.5 km south west from the village; Area 25 × 50 m (100×50 m), 11°06'42.0"N, 013°57'40.7"W, 22 Nov 2013, *C. Couch & F. Fofana CBG BOULCI* • Saberb Boulere (3 km more or less N of Boulere), 11°8'58.1"N, 013°56'54.8"W, 22 Nov 2013, *K. Guilavogui CBG BOUL01*. These sight observations are related to the fertile specimen cited, extracted from the Wet Tropics Africa database at the Royal Botanic Gardens, Kew (see Gosline et al. 2023a). No other species of *Sabdariffa* was recorded on the survey (see below under Conservation Status).

##### Etymology.

Noun in apposition for the town of Sangaredi, a centre of bauxite mining in Guinea, in the vicinity of the area in which the species is restricted based on current information.

##### Distribution and ecology.

Restricted to bowal grassland (tree free) plateau habitats with occasional savannah woodland patches (with *Strychnos spinosa* Lam. (Loganiaceae), *Hymenocardia acida* Tul. (Phyllanthaceae), *Piliostigma thonningii* (Schumach.) Milne-Redh. (Leguminosae), *Crossopteryx febrifuga* (Schumach.) Milne-Redh. (Rubiaceae), *Nauclea latifolia* Sm. (Rubiaceae) and gallery forest (e.g. *Napoleonaea vogelii* Hook. & Planch. (Lecythidaceae), *Uapaca heudelotii* Baill. (Phyllanthaceae), *Tiliacora leonensis* (Scott Elliot) Diels (Menispermaceae), *Malouetia heudelotii* A. DC. (Apocynaceae), *Mostuea hirsuta* (T. Anderson ex Benth. & Hook. f.) Baill. (Gelsemiaceae), *Geophila obvallata* (Schumach.) Diedr. (Rubiaceae), *Bolbitis gemmifera* (Hieron.) C. Chr. (Dryopteridaceae), on bauxitic substrate, rainfall c. 1,100 mm p.a.; 200–300 m alt. The species is deduced to be an annual because of the impoverished root systems and absence of perennating structures detected in the specimens collected.

##### Phenology.

Fruiting and flowering in late November.

##### Conservation status.

The species can be locally common (25% cover recorded in one plot 23 Nov 2013, K. Guilavogui and L. Lopez Poveda *CBG Z5L02*) and frequent, recorded at nine different sites over the four-day survey of the Sangaredi area in 2013 (see additional records above). Indeed, of the 973 specimen linked records made on this survey, nine, nearly 1%, were of *Sabdariffa sangaredi*. The type gathering consists of several individuals per sheet, but information from the additional records cited above suggests that many hundreds of plants, if not thousands, were in the area surveyed.

##### Discussion.

The collector of the type specimen speculates that it is likely to occur on nearby plateau that have not yet been botanically surveyed (Guilavogui pers. obs. to Cheek, Oct 2025). Yet this is not yet supported by evidence and the IUCN advocate adopting the precautionary principle in making conservation assessments (IUCN 2012). Further, the Sangaredi area is heavily impacted by open cast mining of bauxite, with 14 million tonnes being exported p.a. (Sangaredi 2025). There are 10 other bauxite mining operations in coastal Guinea and in total >100 million tonnes are exported p.a. with the totals rising steadily year on year (Nandi 2023). This poses a threat to *Sabdariffa sangaredi* and other threatened species of bauxitic bowal habitats in the area and is likely to already have resulted in the loss of many plants and much habitat of this species from previous mining. Open cast mining is a major source of income for Guinea (Thapar 2016) and is set to continue and increase in the future. Plotting the eight points known for this species (see above) with georeferences available on Google Earth Pro shows that all still have intact habitat for the moment, but that between May and December 2023, terrain was cleared in preparation for mining in the Kagnaka area, which is only 1.4 km from two of the sites for *S. sangaredi*. Moreover, these two sites are in an area with cleared grid lines (clearly visible in Google Earth imagery), suggesting that it has been prospected for bauxite and might also be mined. Similarly, sites for the species N of Boulere are only c. 1.5 km from a new mining area opened since January 2016 (Google Earth Pro time slider function). New areas for mining are steadily being opened up as older areas are exhausted by surface strip mining, the bauxite layer in Guinea being close to the surface and 8–10 m deep (Nandi 2023). The mined areas do not support the natural habitat that formerly existed. The bowal habitat of the species has become severely fragmented due to losses from bauxite mining.

The extent of occurrence (EOO) for *Sabdariffa sangaredi* was calculated as 96 km^2^, and the area of occupation (AOO) as 24 km^2^ using GeoCAT. Since the EOO is below the threshold for Critically Endangered status, and there are clear threats from bauxite mining, projected to result in a decline in EOO, AOO, quality of habitat, and number of mature individuals, we here assess the species as Critically Endangered (CR B1 a,b(i-ii+v)). We plan to contact Compagnie des Bauxite de Guinée (CBG) to alert them to the existence of this species in the area which they manage, provide information to enable the identification of plants, and to request that they take measures to minimize risks to the species, including a public information campaign. It is to be hoped that further surveys might find this species to be more widespread and common, and less threatened.

The type specimen was initially identified as *Hibiscus scotellii* Baker f. by the last author. All the records of this species were made and material collected as part of an Environmental Impact Assessment for the bauxite mining company CBG. The variation in height of plants of this species can be attributed to variation in soil depths and water availability, plants being smaller where depth and water availability are lower than elsewhere.

*Sabdariffa sangaredi* is distinctive for the deeply trilobed leaves that subtend the flowers, succeeding entire ovate or oblong leaves at the base of the main stem (Fig. 1A). This is the inverse of the usual condition in the genus where lobed leaves occur towards the base of the stem and entire leaves above them (Hanes et al. 2024). The lateral leaf lobes extend at almost 90 degrees from the axis of the larger median lobe (Figs 1, 2). The absence of uniformly stellate hairs on stem and calyx lobes, and the presence on the stem of a longitudinal hair band of minute simple hairs, separate it immediately from *S. sineaculeata* and *S. scotellii* even in the absence of reproductive material. In many respects, the species resembles *S. aspera* (Hook. f.) Mwachala and R.L. Barrett, which also has a stem hair band and similar seed surfaces. However, that species has aculeate stems (aculeae are defined as being >1.5 mm long by Barrett et al. (2025), the aculeae being antrorse, while the non-aculeate hairs on *S. sangaredi* are <0.5 mm long and retrorse or patent. Furthermore, all the West African material of *S. aspera* has 5-lobed leaves subtending the flowers and on the vegetative stem, unlike *S. sangaredi* (3-lobed). Moreover, in *S. aspera*, the involucellular bracteoles are free or scarcely united at the base (strongly united in *S. sangaredi*) and the calyx is usually twice as long as the involucellar bracts (subequal in *S. sangaredi*). Both species, together with *S. sineaculeata*, have red, fleshy, thickened bases to the calyx bristle hairs.

*Sabdariffa aspera* is widespread in West Africa. It has been recorded in bowal habitats on other substrates (sandstone and iron ore, usually at submontane elevations) in Guinea, but not on bauxitic bowal at low elevations (the habitat of *S. sangaredi*). In contrast, in Guinea, *S. scotellii* is mainly restricted to granite inselberg habitats.

#### Sabdariffa kounounkan

Taxon classificationPlantaeMalvalesMalvaceae

Cheek sp. nov.

8D8510F8-7FD3-53E0-A837-2B3492D3792D

urn:lsid:ipni.org:names:77376561-1

Figs 3, 4

##### Diagnosis.

*Sabdariffa kounounkan* is most closely similar to *S. sineaculeata* (F.D. Wilson) Mwachala and R.L. Barrett of Ghana-Nigeria, differing in the deciduous (vs persistent) stipules; the involucellar bracts 9–13 mm long united into a short tube at base (vs free, 6–10 mm long); the leaf-blades of the primary axis transversally elliptic in outline, 5(−7) palmately lobed by 1/3, >80% covered in softly pubescent stellate hairs (vs orbicular or ovate, 3(−5)-lobed by 2/3, <10% covered in stellate hairs).

In not having a single nectar gland on the abaxial leaf surface, but constantly three (one each at base of midrib and the adjacent lateral pair of ribs), *Sabdariffa kounounkan* is unusual among the African species of *Sabdariffa* and similar in this respect only to *S. sineaculeata* and *S. scotellii* (Baker f.) Mwachala and R.L. Barrett. However, *S. diversifolia* (Jacq.) McLay and R.L. Barrett and *S. mastersiana* (Hiern) Mwachala and R.L. Barrett, also sometimes have three nectar glands, but are otherwise so morphologically different that they are unlikely to be confused.

**Figure 3.**
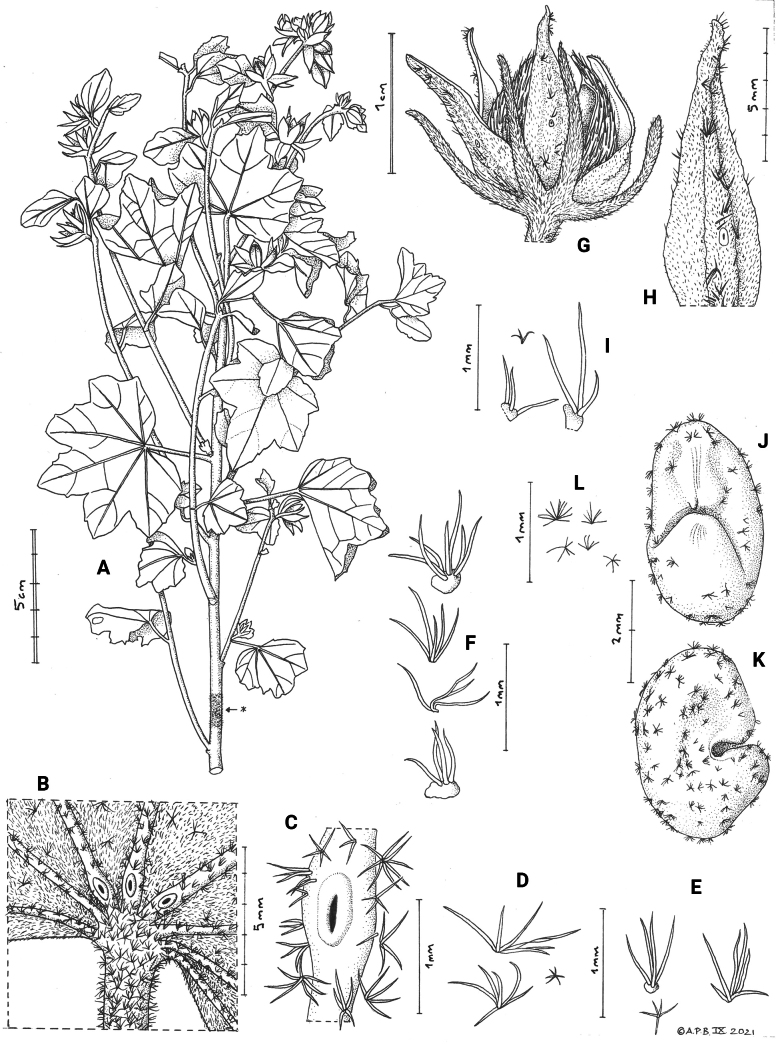
*Sabdariffa kounounkan* Cheek, sp. nov. **A**. Habit, fruiting stem (* = hair details); **B**. Abaxial base of leaf showing nectar glands; **C**. Detail of B; **D**. Hairs of abaxial leaf; **E**. Hairs of adaxial leaf; **F**. Stem; **G**. Hair range fruit, lateral view; **H**. Sepal, abaxial view; **I**. Calyx lobe hair range; **J**. Seed, plan view of radicular and chalazal ends; **K**. Seed, lateral view; **L**. Detail of seed surface hairs. All from *X.M. van der Burgt et al. 2255*. Drawn by Andrew Brown.

##### Type.

Republic of Guinea. • Forécariah Prefecture, southern plateau of Kounoukan Massif, 09°32'53.1"N, 012°51'17.6"W, 3 Feb 2019 (fl, fr), *X.M. van der Burgt P.M. Haba, Konomou, & Xanthos 2255* (holotype: K barcode K000593394; isotype: HNG).

##### Description.

Deciduous ***perennial shrubs*** to 1.2 m tall, stems woody, branched. ***Stems*** of main axis, with internodes 2–3 cm long, c. 4 mm diam. (c. 30 cm from apex); lateral branches arising from almost every node, ascending, 10–15 cm long, proximal internodes c. 4 cm long, contracting to 0.5 cm long distally; stems densely and softly pubescent, ± completely covered with golden to pale brown stellate hairs, hairs 3–7(−10)-armed, arms erect, distally spreading, 0.4–0.7(−1.3) mm long, hair bases swollen, red, subglobose, 0.2–0.3 mm diam., simple hairs and aculeae absent. ***Stipules*** caducous, not seen. ***Leaves*** of primary axis larger than those of lateral branches, spirally inserted; ***petioles*** terete 6–22(−31) × 1–1.5 mm, indumentum as that of the stems; blade transversely elliptic in outline, 5(−7)-palmately lobed by up to a third the radius of the blade, 35–52 × 45–65 mm, median lobes of primary-axis leaves triangular, 12–22 × 15–20 mm, apex glandular, central lobes with (1−)2 shallow dentate teeth per side; leaf blades of lateral branches 3(−5)-lobed, the proximal leaves the same shape as those of the primary axis but c. 2.5 × 3 cm, the distal leaves becoming lanceolate, with reduced lateral lobes, and smaller, 1.4–1.9 × 0.7–1.8 cm; base of blade shallowly cordate, sinus c. 90–120°, 0.5–0.8 mm deep (main axis leaves); abaxial surface of blade white to pale green, nerves 5(−7)-palmate, raised, pale-brown, midrib and the two flanking nerves each with a nectar gland, nectar glands inserted c. 1 mm above petiole, longitudinally elliptic, c.1 × 0.5 mm, white, glabrous, the aperture longitudinal, narrowly elliptic, 0.25–3 mm long; central 3 nerves c. 0.3–0.4 mm wide, ± moderately densely covered in 3–6-armed stellate hairs, hairs bases subglobose, arms stout, yellow, spreading, 0.4–0.75 mm long; intercostal areas completely covered in minute white stellate hairs, arms erect, 0.1–0.2 mm long; mixed with sparse larger stellate hairs as on the nerves, adaxial surface with indumentum similar, but hairs sparser and larger. ***Flowers*** solitary, axillary on the distal nodes of the lateral branches, buds narrowly elliptic c. 1.8 × 0.5 cm; at anthesis c. 3–3.5 cm diam., yellow, with purple-brown centre; peduncles-pedicels 3.5–5.5 × 1.5 mm, articulated at base, indumentum as stem. ***Involucellar bracts*** of the epicalyx 6–8, entire not bifurcate, plano-convex in section, the abaxial surface convex, subulate 9(−13) × (0.9−)1.2–2 mm, even in width, but widening at base and forming a short tube 1–1.2 mm long, apices obtuse and slightly reflexed, often with a red-glandular patch, epidermis drying dull pink or flesh-coloured, 50–70%, covered in 3–6-armed stellate hairs, arms erect, slightly spreading, gold or white, 0.25–0.5 mm long evenly spread over entire surface, covering epidermis. ***Calyx*** exceeding epicalyx, (11.5−)12–16 cm long, divided by c. ⅔ into 5 sepals, the united part cupular, 4–5 × 10–12 mm; sepals lanceolate, 11–14 × 3–3.7 cm, apices acute, abaxial surface with midrib flat, raised, c. 1 cm broad, not fleshy; marginal nerves sometimes inconspicuous, nectary gland inserted 2–5.5 mm from the base either in distal part of the united calyx or the proximal part of the free sepal, nectary white, aperture circular or elliptic, c. 0.7 mm long; indumentum minutely soft white stellate hairy, dense, covering the entire surface, hairs 3–6-armed, arms filamentous, c. 0.1 mm long; nerves with larger, sparse, golden, stiff, simple, 2–3-fid and rarely stellate hairs, 0.5–1 mm apart, on globose, thickened bases c. 0.2 mm diam., arms thick, erect or slightly spreading, 0.4–1.1(−2) mm long. ***Petals*** 5, yellow, internally with purple-brown base, c. 1.5–1.8 × 1.2 cm, abaxial surface in bud densely simple hairy, hairs crisped, erect, c. 0.1 mm long, covering 30–50% of the surface.

**Figure 4.**
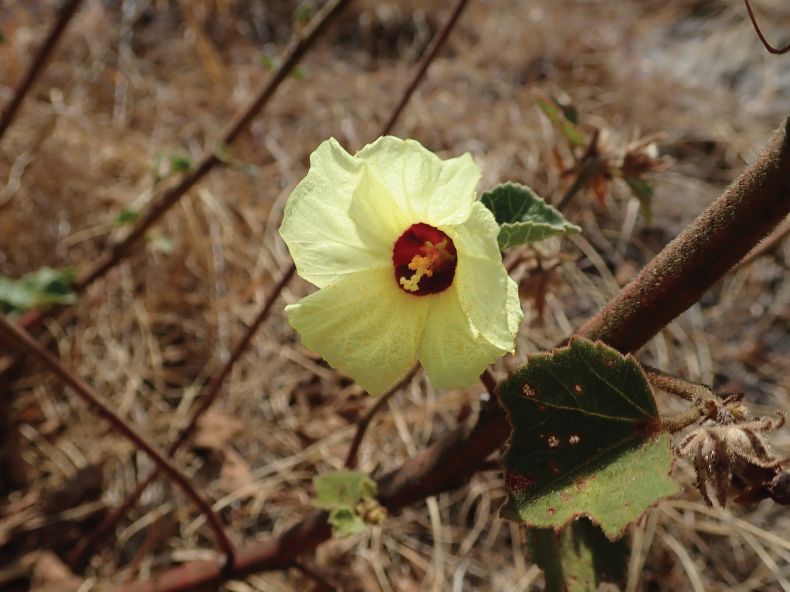
*Sabdariffa kounounkan* Cheek, sp. nov. Photo of type specimen in flower, in habitat at Kounounkan, by Xander van der Burgt.

Androecium and gynoecium unknown (material too scarce to dissect). ***Capsules*** 5-valved, 12–14 × 10–12 mm, the valves 4 mm wide, widest in the distal half, spreading after dehiscence, apex rostrate, rostrum 1–2 mm long, valves c. 3 mm deep, inner surface glabrous, glossy lemon-yellow, outer surface densely covered in golden straight, appressed, thick, simple setose hairs 2–3 mm long, soon falling from the distalmost part leaving a naked glossy, pale-brown surface. ***Seeds*** subreniform to orange-segment shaped, c. 4 × 2.2 × 2.4 mm, the ventral hilum sinus slit-like, c. 1 mm deep, glabrous; surface deep purple-black, stellate hairs white, 15–25% cover, 0.2–0.5 mm diam., 5(−20)-armed, arms filamentous.

##### Etymology.

The specific epithet is a noun in apposition that honours the Kounounkan area of Guinea which harbours the largest number of the site-specific global endemics in the nation.

##### Distribution and ecology.

*Sabdariffa kounounkan* appears endemic to the sandstone table mountain of the southernmost Fouta Djalon highlands at Kounounkan, Guinea. It occurs in fissures in the bedrock, in sparsely wooded (bowal) grassland at 990 m alt. This is perhaps the last sandstone table mountain with pristine areas remaining in the country. The lead collector, van der Burgt (pers. obs. to Cheek 2025) states that although one collection was made, the species was common and widespread at the site, with potentially thousands of plants present. Some plants were in areas that had been burned artificially (fires set by people are almost ubiquitous in Guinea) and appeared to have survived, so the species may not be as sensitive to fire as some other of the rare sandstone table mountain species of Kounounkan appear to be.

Associated plant species observed with *Sabdariffa kounounkan* included *Memecylon afzelii* G. Don (*van der Burgt 2252*, Melastomataceae), *Tricalysia okelensis* var. *pubescens* Aubrév. & Pellegr. ex Keay (*van der Burgt 2251*, Rubiaceae), *Keetia kounounkan* Cheek (*van der Burgt 2262*, Rubiaceae; Simbiano et al. 2025), *Ternstroemia guineensis* Cheek (*van der Burgt 2258*, Pentaphylacaceae), *Warneckea fascicularis* (Planch. ex Benth.) Jacq.-Fél. (*van der Burgt 2249*, Melastomataceae), *Psorospermum febrifugum* Spach (*van der Burgt 2253*, Hypericaceae)), *Ficus ovata* Vahl, *Cailliella praerupticola* Jacq.-Fél. (*van der Burgt 2263*, Melastomataceae), *Glenniea africana* (Radlk.) Leenh. (Sapindaceae), *Kotschya uniflora* (A. Chev.) Hepper (Leguminosae), *Keetia mannii* (Hiern) Bridson, and *Keetia susu* Cheek (both Rubiaceae).

##### Phenology.

*Sabdariffa kounounkan* flowers and fruits in early February. However, the collection notes indicate that at that time, most plants were without leaves and flowers. It is likely therefore that this was the tail end of flowering that likely began at the start of the dry season in October. It is likely that the species is a perennial and that it survives the dry season in a leafless, dormant state.

##### Conservation status.

Here we follow the provisional assessment made for *Keetia kounounkan* (Simbiano et al. 2025), also a shrub known from a single collection and collected at the same time and place as *Sabdariffa kounounkan* (the two specimens are separated by only 6 numbers of plants collected), by the same team. The extent of occurrence (EOO) of *S. kounounkan* is estimated to be no greater than 16 km^2^, based on the area of the southern plateau of the Kounounkan Massif (see Discussion) from which the only known collection and observations have been made. Its area of occupancy (AOO) across the plateau area is also likely to be highly restricted but may narrowly exceed 10 km^2^. The plateau is considered to represent a single location threatened by dry-season bushfires set by cattle herders. As a result of this threat, the species is inferred to be undergoing a continuing decline in habitat quality. The number of mature individuals cannot be reliably estimated, but it is suspected that the true value may exceed 1,000.

Given the availability of other similar submontane habitats in neighbouring Kindia and Dubréka prefectures, it is possible that this species occurs at other sites; however, it has not yet been reported from collecting trips to neighbouring plateaus. Pending more precise data on its distribution and population size and adopting a precautionary approach on the basis that its distribution may prove to be highly restricted, *Sabdariffa kounounkan* is here provisionally assessed as Critically Endangered (CR) B1ab(iii), following IUCN criteria. Further survey work is essential to refine this conservation assessment; for example, confirmation of its presence on other plateaus may permit assessment at a lower category of extinction risk, though it would likely remain threatened.

Seeds of *Sabdariffa kounounkan* are held at the national seed bank of the Republic of Guinea at the National Herbarium of Guinea (HNG) and also the Millenium Seed Bank Partnership at Wakehurst Place, Sussex, UK (van der Burgt pers. obs. to Cheek 2025).

##### Discussion.

In Wilson (1999), our Guinea material, since it lacks aculeate stems, bifid involucellar bracts and a fleshy calyx, yet has foliar nectaries and subulate, involucellar bracteoles, c. 2 mm wide at base, apex pointed, calyx both finely stellate pubescent and with simple to stellate stiff bristles on enlarged red bases, keys to *Sabdariffa sineaculeata* (F.D. Wilson) Mwachala and R.L. Barrett. The two species can be separated using features in the diagnosis and in Table 1 below.

**Table 1.** Characters separating *Sabdariffa kounounkan*, *S. sangaredi*, and *S. sineaculeata*. Data on the latter species are from Wilson (1999) and specimens at Kew.

	*Sabdariffa kounounkan*	*S. sangaredi*	*S. sineaculeata*
Indumentum of adaxial leaf surface	Dense, hairs covering > 70% of surface, soft, stellate, arms 0.1–0.2 mm long	Very sparse (<5% cover) minute simple to stellate hairs c. 0.1 mm long	Sparse, hairs covering <10% of surface, stellate
Stipules	Fugacious	Deciduous, subulate to linear-oblong, 0.75–1.75 mm long	Persistent, 2–4 mm long
Leaf-blades of primary axis	Transversely elliptic in outline; 5–7-lobed by up to 1/3 radius	Triangular in outline; 3(−5) lobed by up to 8/10 radius; lateral lobes at c. 90 degrees from the median lobe, median lobe 10–20% longer and broader than the laterals, sometimes with a secondary lobe	Ovate or orbicular in outline; 3-lobed by 2/3 radius
Calyx: epicalyx length ratio	± 1:1	± 1:1 (calyx slightly longer)	± 2:1
Epicalyx bract length; nerves conspicuous or not	9–13 mm; nerves absent or inconspicuous	12.5–16 mm; midrib nerve conspicuous only	6–10 mm; several-nerved
Calyx proportions united: free	1:2	1:7–1:9	1:3–1:5
Geographic range	Kounounkan, Forecariah Prefecture, Guinea	Sangaredi, Boké Prefecture, Guinea	Ghana to Nigeria
Seed-coat surface	White stellate hairy	Subglabrous; longitudinal ridges and minute convex structures in rows	Subglabrous

### New endemics of sandstone habitats in Guinea

The Ordovician sandstone plateau of the Fouta Djalon highlands area (sensu lato, Couch et al. 2019) is probably the single most important source of endemic species in Guinea, of which *Sabdariffa kounounkan* is only the most recent to be published. All but one (*Feliciadamia* Bullock, Melastomataceae) of Guinea’s endemic genera appear confined to this substrate (see Introduction). New species and even new genera to science additional to those mentioned above are steadily being discovered and published from habitats of Guinean sandstone:

From sandstone cliffs: *Kindia gangan* Cheek (Rubiaceae), *Trichanthecium tenerium* Xanthos (Poaceae), *Benna alternifolia* Burgt & Ver.-Lib. (Melastomataceae), *Virectaria stellata* Cheek et al. (Rubiaceae) (Cheek et al. 2018a; Xanthos et al. 2020; van der Burgt et al. 2022; Simbiano et al. 2024).

From sandstone bowal habitats (including fissures): *Eriocaulon cryptocephalum* S.M. Phillips & Mesterházy (Eriocaulaceae), *Gladiolus mariae* Burgt (Iridaceae), *Tephrosia kindiana* Haba et al. (Leguminosae) (Phillips and Mesterházy 2015; van der Burgt et al. 2019; Haba et al. 2023).

From waterfalls and rapids on sandstone: *Inversodicraea tassing* Cheek, *Inversodicraea koukoutamba* Cheek, *Saxicolella futa* Cheek (all Podostemaceae), *Ctenium bennae* Xanthos (Poaceae) (Cheek et al. 2019c; Cheek et al. 2022; Xanthos et al. 2021).

From evergreen forest (patches and gallery): *Talbotiella cheekii* Burgt (Leguminosae), *Keetia susu* Cheek (Rubiaceae, also bowal), *Ternstroemia guineensis* Cheek (Ternstroemiaceae), *Keetia kounounkan* Cheek (Rubiaceae) (van der Burgt et al. 2018; Cheek et al. 2018b; Cheek et al. 2019b; Simbiano et al. 2025).

The greatest concentration of these endemics, over 70% of those listed above, has been found at Kounounkan, a table mountain at the southern extremity of the sandstone plateau, which extends close to the border with Sierra Leone. This is the type and currently the only known locality of *Sabdariffa kounounkan*. Kounounkan was designated as a TIPA (Tropical Important Plant Area) in 2019 (Couch et al. 2019 following the standard of Darbyshire et al. 2017) and the area of 39.5 km^2^ is set to become a formally protected area. Of the 22 TIPAs in Guinea, it was recorded as having the highest number of strictly endemic species (31), with seven globally unique species recorded (Couch et al. 2019: 124–132). Subsequently, some of these species have been found elsewhere such as the adjoining Benna Plateau, or in the Mt Gangan TIPA, but at the same time, additional new endemic and near endemic species have been published from Kounounkan and nearby sandstone plateaus (listed above).

The plateau ranges from c. 100–1,180 m alt. and is the most ecologically intact surviving area of threatened sandstone habitats, which elsewhere in the wider Fouta Djalon have been destroyed or largely degraded by agriculture, especially livestock grazing and trampling and the setting of frequent fires to support the raising of livestock. Consequently, almost all these endemics have been assessed as highly threatened and several, perhaps many of the historically documented species, may now be globally extinct. Of the 25 endemic species of Guinea which have not been seen alive in over 60 years (Couch et al. 2019), the majority are thought to be from sandstone, but so far only one, *Inversodicraea pygmaea* G. Taylor, has had targeted searches at the correct season and been assessed as possibly extinct (Cheek and Magassouba 2018; Cheek 2018).

## Conclusion

Further botanical inventories in under-collected areas of Guinea are required if we are to uncover the remaining plant species that are yet unknown to science and to access their conservation status. This is urgent, as three out of every four species described as new to science are assessed as threatened at the point of publication (Brown et al. 2023). Remaining intact natural habitat in Guinea is being steadily developed at the expense of indigenous threatened biodiversity, with agriculture and mining among the major threats. Once a species appears as threatened on the Red List, there are greater possibilities of attracting attention from authorities and land managers to protect, rather than destroy, such species and their habitats. It may be possible to include at-risk species in Important Plant Areas or TIPAs (Couch et al. 2019) when the possibility of acquiring protected area status is increased. It is also advisable to establish in detail the risks that such species face in their habitats to better address them (Couch et al. 2025) via action plans, which can be developed to mitigate these risks and attempt to reverse the trend towards extinction of such species (Couch et al. 2022). Public sensitisation programmes are advised as an insurance policy. Seed conservation, and propagation in nurseries and safeguarding in botanic gardens is advisable. Such actions may mitigate the risk of further species following in the footsteps of *Inversodicraea pygmaea* towards likely global extinction.

## Supplementary Material

XML Treatment for Sabdariffa sangaredi

XML Treatment for Sabdariffa kounounkan

## Additional information

### Use of AI

No use of AI was reported.

### Author ORCIDs

Sekou Magassouba https://orcid.org/0000-0002-8059-7788

Charlotte Couch https://orcid.org/0000-0002-5707-9253

Martin Cheek https://orcid.org/0000-0003-4343-3124

### Data availability

All of the data that support the findings of this study are available in the main text.
